# Mechanistic insight into the protective effects of fisetin against arsenic-induced reproductive toxicity in male rats

**DOI:** 10.1038/s41598-023-30302-x

**Published:** 2023-02-22

**Authors:** Muhammad Umar Ijaz, Saqlain Haider, Arfa Tahir, Tayyaba Afsar, Ali Almajwal, Houda Amor, Suhail Razak

**Affiliations:** 1 Department of Zoology, Wildlife and Fisheries, University of Agriculture, Faisalabad, Pakistan; 2 Department of Community Health Sciences, College of Applied Medical Sciences, King Saud University, Riyadh, Saudi Arabia; 3 Department of Obstetrics, Gynecology and Reproductive Medicine, Saarland University Clinic, Homburg, Germany

**Keywords:** Biochemistry, Biomarkers

## Abstract

Arsenic is one of the most hazardous environmental contaminants, which adversely affects the dynamics of male reproductive system. Fisetin (FIS) is a bioactive flavonoid, which is known to exert strong antioxidative effects. Therefore, the current research was planned to evaluate the alleviative efficacy of FIS against arsenic-induced reproductive damages. Forty-eight male albino rats were divided into 4 groups (n = 12), which were treated as follows: (1) Control, (2) Arsenic-intoxicated group (8 mg kg^−1^), (3) Arsenic + FIS-treated group (8 mg kg^−1^ + 10 mg kg^−1^), and (4) FIS-treated group (10 mgkg^−1^). After 56 days of treatment, the biochemical, lipidemic, steroidogenic, hormonal, spermatological, apoptotic and histoarchitectural profiles of rats were analyzed. Arsenic intoxication reduced the enzymatic activities of catalase (CAT), superoxide dismutase (SOD), glutathione peroxidase (GPx) and glutathione reductase (GSR), in addition to glutathione (GSH) level. Conversely, the levels of thiobarbituric acid reactive substance (TBARS) and reactive oxygen species (ROS) were increased. Moreover, it escalated the level of low-density lipoprotein (LDL), triglycerides and total cholesterol, while declining the level of high-density lipoprotein (HDL). Furthermore, steroidogenic enzymes expressions, 3β-hydroxysteroid dehydrogenase (HSD), 17β-HSD, steroidogenic acute regulatory protein (StAR), cholesterol side-chain cleavage enzyme (CYP11A1) and 17α-hydroxylase/17, 20-lyase (CYP17A1), were found to be reduced, which brought down the level of testosterone. Besides, the levels of gonadotropins (LH and FSH) were decreased. Additionally, a decline in sperm mitochondrial membrane potential (MMP), motility, epididymal sperm count and hypo-osmotic swelling (HOS) coil-tailed sperms was observed, whereas the dead sperms and structural damages (head, midpiece and tail) of sperms were escalated. Moreover, arsenic exposure up-regulated the mRNA expressions of apoptotic markers, namely Bax and caspase-3, whereas lowered the expression of anti-apoptotic marker, Bcl-2. In addition, it induced histoarchitectural changes in testes of rats. However, FIS treatment resulted in remarkable improvements in testicular and sperm parameters. Therefore, it was inferred that FIS could serve as a therapeutic candidate against arsenic-generated male reproductive toxicity attributing to its anti-oxidant, anti-lipoperoxidative, anti-apoptotic, and androgenic efficacy.

## Introduction

Arsenic is a hazardous environmental contaminant, which is considered as a king of poisons. It is ranked at the top in the list of hazardous substances by Agency for Toxic Substances and Disease Registry (ATSDR) on the basis of the extent of toxicity, prevalence and exposure to humans^1^. Arsenic is an endocrine-disrupting chemical that has tendency to mimic the action of hormones in the body. Agricultural and industrial effluents are the major sources of arsenic exposure to the humans^2^. It can enter into the body via food, water and air; however, drinking the arsenic-contaminated water is the main source of arsenic exposure. Arsenic level is increasing in drinking water due to anthropogenic activities, including smelting, mining, burning of fossil fuel, discharge of industrial effluents as well as use of arsenic-containing fertilizers and pesticides^3^. According to WHO, the permissible arsenic level in drinking water is 10 µg/L^4^. It is indicated that this level has been surpassed in several countries and approximately 100 million people are at the risk of arsenic exposure by drinking the contaminated water^5^. The arsenic intoxication is reported to cause a wide range of damages due to the generation of oxidative stress, that disrupts the macromolecules, such as proteins, lipids and DNA^1^.

The cardiac abnormalities, muscular cramps, facial edema, diarrhea and vomiting are the signs of acute arsenic toxicity, whereas the chronic symptoms include, stomach pain, anorexia, disturbance in the bowel habits (i.e., constipation and diarrhea) and lethargy^6^. The testicular and epididymal tissues are considered as one of the important target sites of arsenic exposure in the body^7^. Ijaz et al.^8^ stated that the testicles are extremely prone to toxicity due to enrichment with polyunsaturated fatty acids (PUFAs) in tissues. Previous studies indicated that arsenic exposure generates toxicity by binding with thiol groups of biological molecules, thereby causing testicular malfunctions. Moreover, it has tendency to exert harmful effects in male reproductive system by decreasing the testosterone level and reducing the activities of antioxidative enzymes^9^. Furthermore, arsenic is observed to reduce the sperm count and damage testicular histology^10,11^. Therefore, it is indispensable to explore some remedial measure to counter the arsenic-instigated testicular toxicity.

Flavonoids are considered as curative compounds due to their potent pharmacological properties; hence, they are extensively used in medicines and food supplements^12^. FIS (3, 7, 3′, 4′-tetrahydroxyflavone) is a bioactive flavonoid, which is extracted from plants possessing nutritional properties^13^. It is present in flowers (chamomile and lime blossom), vegetables (cucumber, onion, lotus root and tomato), fruits (apple, kiwi, persimmon, peach, red grapes, white grapes and strawberries) and teas (black, green red and Ceylon tea) as well as in Anacardiaceae plants^14^. The assessment of FIS pharmacokinetics in rats revealed that it has high and rapid biotransformation capacity, which makes it a suitable candidate for the pharmaceutical industry^15^. FIS exerts anti-cancerous, neuroprotective, anti-inflammatory, and ROS-scavenging effects^16^. FIS is highly lipophilic. This property enables it to move across the cell membranes easily and confers it the antioxidative potential^17^.

FIS was reported to be one of the strongest antioxidants, when its efficacy was compared with several other flavonoids^18^. In another study, electrochemical dynamics of FIS were assessed and it was revealed that FIS carries remarkable antioxidant potential due to its molecular structure and hydroxyl bond dissociation energy^19^. Considering the aforementioned pharmacological properties of FIS as well as hazardous effects of arsenic on male reproductive system, present study was designed to assess the protective effect of FIS against the arsenic-induced male reproductive damage by analyzing the biochemical, steroidogenic, hormonal, and semen indices of rats. In addition to it, the apoptotic profile and testicular histopathology were analyzed.

## Methods

### Animal ethics approval

Animals handling and treatments were supervised by ethical committee of UAF Pakistan, which is according to European Union of animal care and experimentation (CEE Council 86/609)-approved protocols. The study was carried out **i**n accordance with ARRIVE guidelines.

### Animals

48 healthy adult male albino rats (150–180 g) were procured from Animal house of UAF Pakistan. All animals were placed in steel cages, in a well-ventilated room at an ambient temperature of 24–26 °C under maintained relative humidity. Throughout the experiment, the animals under trial were provided with diet pellets and tap water.

### Experimental design

All 48 rats were equally divided into four groups (n = 12) and they were provided with following treatments: (1) Control group was provided with saline (0.9% NaCl) by oral gavage, (2) Arsenic-treated group was orally administered with 8 mg kg^−1^ of arsenic, (3) Arsenic + FIS-treated group was orally provided with the 8 mg kg^−1^ of arsenic as well as 10 mg kg^−1^ of FIS, and (4) FIS treated-group received the 10 mg kg^−1^ of FIS by oral gavage. The treatments were given for 56 days since it takes approximately 56 days to complete one spermatogenic cycle in rats.

The 8 mg kg^−1^ of arsenic dose was decided as per the previous study of Momeni et al.^20^, while 10 mg kg^−1^ of FIS was selected according to an earlier investigation by Prasath and Subramanian^21^. After completion of experimental period, rats were slaughtered under mild anesthesia by 60 mg/kg of ketamine and 6 mg/kg of xylazine. Blood samples were taken and plasma was separated by centrifugation at 322 g for 20 min and preserved at −20 °C for hormonal analysis. The testicular tissues were separated for further analysis. The epididymis was preserved for the assessment of epididymal sperm count. After washing with ice cold saline, left testes were kept in a vial containing 10% formaldehyde for histopathological analysis and right testes were packed in zipper bags and stored at −80 °C to analyze the biochemical profile.

### Biochemical analysis

The activity of CAT was estimated according to method of Aebi^22^. The SOD activity was measured in accordance with the procedure of Kakkar et al.^23^ with slight modification. The GPx activity was evaluated by using a method given by Rotruck et al.^24^. GSR activity was evaluated by the procedure of Carlberg and Mannervik^25^. GSH level was determined by applying the technique introduced by Jollow et al.^26^. TBARS level was gauged by a method explained by Wright et al.^27^. Level of ROS was analyzed as per the procedure stated by Hayashi et al.^28^.

### Lipid profile analysis

The evaluation of total cholesterol, triglycerides, HDL, and LDL was carried out from plasma by using the commercial kits (Diagnostic Product Corporation, Los Angeles, USA).

### Real-time quantitative polymerase chain reaction (qRT-PCR)

mRNA expressions of steroidogenic enzymes (17β-HSD, 3β-HSD, StAR, CYP11A1, and CYP17A1) and apoptotic markers (Bcl-2, Bax, and caspase-3) were assayed using RNeasy reagent for isolating the total RNA, whereas NanoDrop-2000 spectrophotometer was used for evaluating the concentration of RNA. Total RNA with the ratio A260/A280 was used for reverse transcriptase PCR. RNA reverse-transcription was conducted by Prime Script™ RT Master Mix kit. qRT-PCR was conducted using SYBR Green. The internal control was β-actin and alterations in gene expressions were determined by 2^-ΔΔCT^. Table 1 depicts the primer sequences^8^.

**Table 1:** Primer sequences for qRT-PCR.

Gene	Primers 5'—> 3'	Accession number	Product size	Temperature
3β-HSD	F: GCATCCTGAAAAATGGTGGC	NM_001007719	135	57
	R: GCCACATTGCCTACATACAC			
17β-HSD	F: CAGCTTCCAAGGCTTTTGTG	NM_054007	161	59
	R: CAGGTTTCAGCTCCAATCGT			
StAR	F: AAAAGGCCTTGGGCATACTC	NM_031558	113	58
	R: CATAGAGTCTGTCCATGGGC			
CYP11A1	F: GTGCTGGTCAAAAGCTGCCA	J_05156.1	144	60
	R: TTCTGTGTGTGCCGTTCTCC			
CYP17A1	F: TTCTCCCCAGACGTGGTCAT	NM_012753	121	58
	R: CGACCAGAGAATTCTTTTCCCT			
Bax	F: GGCCTTTTTGCTACAGGGTT	NM_017059.2	119	58
	R: AGCTCCATGTTGTTGTCCAG			
Bcl-2	F: ACAACATCGCTCTGTGGAT	NM_016993.1	103	57
	R: TCAGAGACAGCCAGGAGAA			
Caspase-3	F: ATCCATGGAAGCAAGTCGAT	NM_012922.2	233	57
	R: CCTTTTGCTGTGATCTTCCT			
β-actin	F: TACAGCTTCACCACCACAGC	NM_031144	135	58
	R: GGAACCGCTCATTGCCGATA			

### Hormonal analysis

ELISA kits were used for evaluating the levels of the LH (Cusabio, CSB-E12654r; 0.3 mlU/ml-60 mlU/mL), FSH (Cusabio, CSB-E06869r; 0.156–10 ng/mL), and plasma testosterone (Cusabio, CSB-E05100r; 0.13–25.6 ng/mL).

## Sperm assays

### Epididymal sperm count

For the evaluation of sperm count, the Neubauer haemocytometer was used and the methodology explained by Yokoi et al.^29^ was followed with few amendments. Epididymal content from rats was taken by incising caudal epididymis and pressing it in petri dish for taking the semen. After that, incubation was conducted for half an hour at 37 °C for liquification. Then, the supernatant was diluted and the 10 µL of this mixture was dropped in counting chamber. Ultimately, the sperm count was observed via light microscopy at 400**×**.

### Sperm motility

Spermatological motility was analyzed using a technique described by Moumeni et al.^30^. Initially, sample was dispensed on the pre-warm (35℃) slide by a pipette. The dilution was made with the 2 mL of Tris buffer solution. 3 various fields were observed via light microscopy for each semen sample and the mean of 3 values was taken as sperm motility.

### Sperm viability

Percentage of dead sperms was estimated according to the technique of Aksu et al.^31^. 2 droplets of Eosin/Nigrosin were dispensed to the semen on warm slides, the sample was properly smeared and air-dried to observe under light microscope at 400**×**. The alive sperms did not absorb the stain; therefore, they appeared unstained. Conversely, the dead sperms were stained. 200 sperms per sample were counted and percentage of dead sperms was calculated.

### Sperm morphological anomalies

Sperm morphological abnormality was evaluated by drying the semen sample (5 μL) in air and Giemsa was used for staining^32^. After half an hour, extra stain on slides was removed and the sperm abnormalities in samples were observed via microscope at 400** × **. 5 fields from each sample were randomly chosen and the various types as well as number of structurally damage sperms were noticed to compute the percentage of head, mid-piece and tail anomalies.

### Hypo-osmotic swelling test

Membranous integrity of sperms was gauged by the eosin/nigrosin staining^33^. The sample (20 μL) was incubated in fructose solution (180 μL) for approximately 15 min, while the osmotic pressure was kept at 80 mOsm/L. Later on, the solution was properly mixed and stained using eosin/nigrosine. 200 sperms were observed via light microscope at 40 × magnification and the percentage of sperms having swollen and non-swollen tails was calculated.

### Sperm MMP

The lipophilic dye, JC-1, was used to differentially mark mitochondria with low and high membrane potential^34^. Standard solutions with 1 μL of JC-1 (1.53 mM) were prepared in DMSO and epididymal semen sample (50 μL) was dispensed in it. Incubation was carried out for half an hour at 37 °C. After that, the slides were prepared from the mixture samples and observed carefully under epifluorescence microscope. The excitation filter and barrier filter were kept at 510–560 nm and 505 nm respectively. Approximately 200 sperms were noticed (1000x) and the midpieces of sperm cells displaying yellowish orange fluorescence were regarded as positive for MMP, whereas green fluorescence exhibited negative MMP.

### Histopathological analysis

For histopathological observation, 10% formaldehyde was used for fixing tissues and ascending grades of alcohol were used for dehydrating. Later on, testicular tissues were embedded in paraffin wax. Slices (5 μm) of embedded tissues were sliced by using microtomes (Thermo, Shandon finesse 325, UK) and later stained with hematoxylin/eosin. These thick sections were observed by Nikon optiphot research microscope (Japan) and Image J2x software package program was used to take the readings.

### Statistical analysis

Data was displayed as Mean ± SEM. Whole data were analyzed by one-way ANOVA followed by Tukey’s test. Minitab software was used for comparative assessment of various groups. Significance level was kept at *p* < 0.05.

## Results

### Effect of arsenic and FIS on biochemical parameters

The role of As and FIS on the activities of antioxidative enzymes and oxidative stress markers is shown in Table 2. Arsenic treatment significantly (*p* < 0.05) lessened the activity of enzymes, namely CAT, SOD, GPx, GSR, and GSH level, whereas raised the level of oxidative stress markers, i.e., TBARS and ROS in arsenic-exposed rats compared with control. However, FIS supplementation with the arsenic considerably elevated the activity of aforementioned antioxidative enzymes, while lowered the level of TBARS and ROS in comparison to arsenic-induced rats. Additionally, non-significant difference was seen among the animals belonging to FIS-treated group and control.

**Table 2:** Role of arsenic and FIS on biochemical parameters.

Groups	CAT (U/mg protein)	SOD (U/mg protein)	GSR (nM NADPH oxidized/min/mg tissue)	GPx (U/mg protein)	GSH (μM/g tissue)	TBARS (nM TBARS/min/mg tissues)	ROS (U/mg tissue)
Control	6.24 ± 0.13^a^	5.24 ± 0.17^a^	3.66 ± 0.16^a^	10.08 ± 1.50^a^	18.69 ± 0.64^a^	14.69 ± 0.41^c^	0.89 ± 0.14^c^
Arsenic	3.54 ± 0.20^b^	3.30 ± 0.11^b^	2.07 ± 0.09^c^	4.17 ± 0.27b	6.59 ± 0.40^c^	25.87 ± 0.12^a^	7.78 ± 0.35^a^
Arsenic + FIS	6.10 ± 0.06^a^	4.99 ± 0.06^a^	2.90 ± 0.15^b^	9.78 ± 0.55^a^	14.69 ± 0.71^b^	16.61 ± 0.55^b^	2.14 ± 0.10^b^
Arsenic	6.29 ± 0.08^a^	5.11 ± 0.57^a^	3.07 ± 0.04^b^	10.49 ± 1.61^a^	18.95 ± 0.68^a^	16.16 ± 0.20^b^	0.88 ± 0.13^c^

Data displayed as Mean ± SEM (12 rats/group). Results of each sub-parameter with varying alphabets are considered significantly different (*p* < 0.05).

### Effect of arsenic and FIS treatments on lipid profile

The alterations in lipid profile followed by the treatment of arsenic and FIS are shown in Table 3. Assessment of lipid profile of the arsenic-intoxicated group revealed that the level of bad quality fats, i.e., LDL, triglycerides and total cholesterol, were significantly elevated, whereas the level of good quality fat, i.e., HDL, was substantially declined in arsenic-administered group compared to control. Nonetheless, co-treatment with arsenic and FIS lessened the levels of LDL, triglyceride and total cholesterol, in addition to increasing the HDL level when compared to arsenic-treated group. However, there was non-significant difference among the FIS (only)-treated and control animals.

**Table 3:** Effect of arsenic and FIS on lipid profile.

Groups	HDL (mg/dL)	LDL (mg/dL)	Triglyceride (mg/dL)	Total cholesterol (mg/dL)
Control	30.48 ± 0.87^a^	3.88 ± 0.55^a^	52.99 ± 1.29^a^	32.47 ± 1.30^a^
Arsenic	10.89 ± 0.66^b^	16.97 ± .85^b^	100.2 ± 2.73^b^	63.20 ± 2.06^c^
Arsenic + FIS	28.92 ± 0.95^a^	5.65 ± 0.51^a^	54.68 ± 1.12^a^	38.88 ± 0.80^b^
FIS	31.92 ± 1.20^a^	3.78 ± 0.22^a^	53.95 ± 1.39^a^	35.73 ± 1.17^b^

Data displayed as Mean ± SEM (12 rats/group). Results of each sub-parameter with varying alphabets are considered significantly different (*p* < 0.05).

### Effect of arsenic and FIS on expressions of steroidogenic enzymes

**Figure 1:**
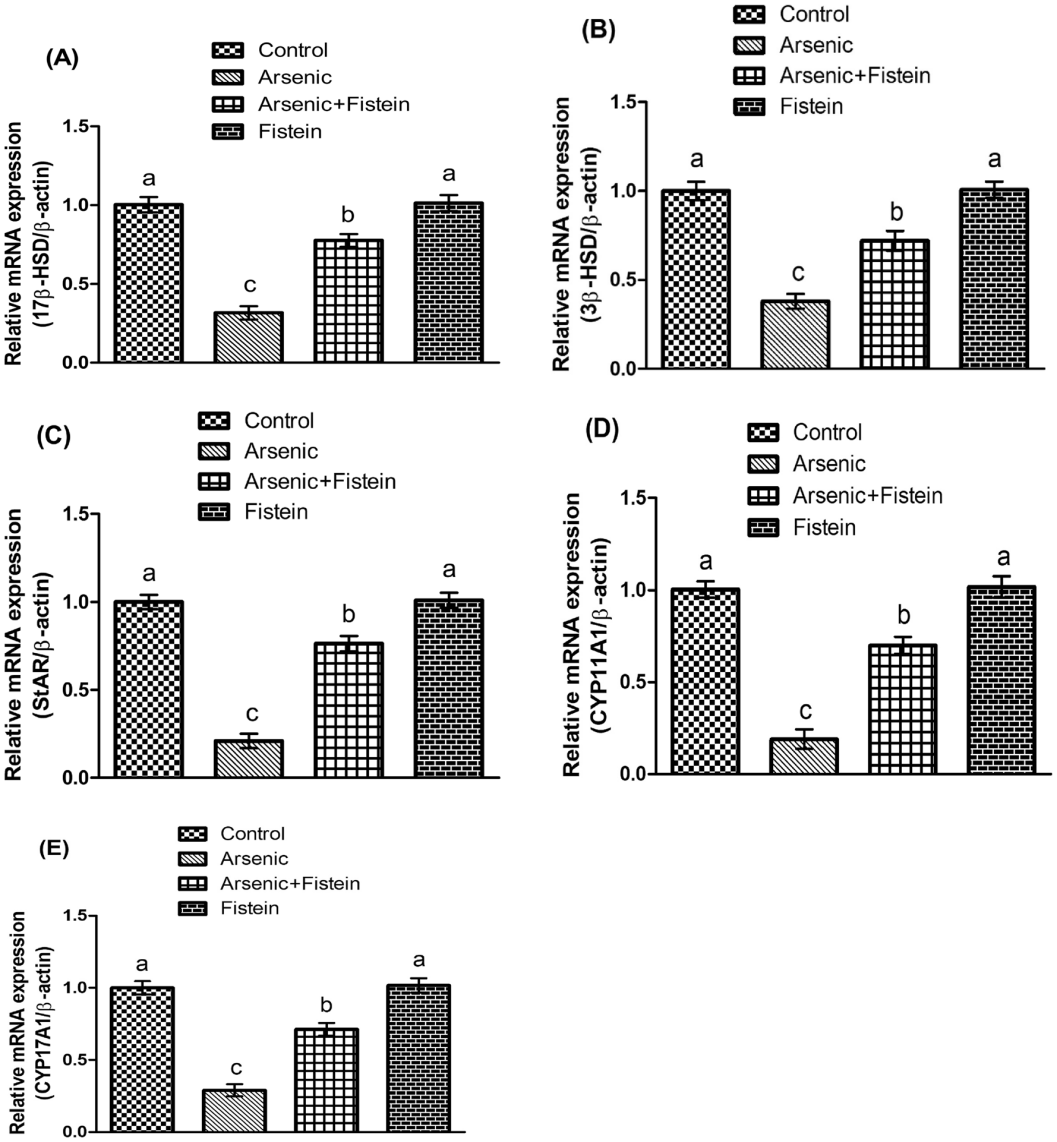
Impact of arsenic and FIS on encoding of (**A**) 17β-HSD, (**B**) 3β-HSD, (**C**) StAR, (**D**) CYP11A1, and (**E**) CYP17A1. Data shown as Mean ± SEM (12 rats/group). Means with dissimilar letters vary significantly and the letters indicate significant difference at *p* < 0.05.

Effect of arsenic and FIS on expressions of steroidogenic enzymes is shown in Fig. 1. Arsenic exposure lessened the expressions of 17β-HSD, 3β-HSD, StAR, CYP11A1 and CYP17A1 as compared with control. Whereas, co-treatment with the rats with arsenic and FIS resulted in the up-regulation of the expressions of above-mentioned enzymes compared to arsenic (only)-treated group. Besides, there was not any notable difference among the FIS-exposed and control rats.

### Effect of arsenic and FIS on hormones

Alterations in the level of hormones following the arsenic and FIS exposure are exhibited in the Table 4. After the administration of arsenic, a noteworthy (*p* < 0.05) decrease was noticed in levels of LH, FSH and plasma testosterone compared to control. However, the co-administration of arsenic and FIS raised the level of these hormones with respect to arsenic-exposed group. In addition to it, the FIS-administered group and control did not depict any significant difference.

**Table 4:** Effect of arsenic and FIS on levels of hormones.

Groups	LH (mIU/mL)	FSH (mIU/mL)	Plasma testosterone concentration (ng/mL)
Control	2.49 ± 0.03^a^	3.23 ± 0.08^a^	6.08 ± 0.07^a^
Arsenic	1.20 ± 0.09^b^	1.65 ± 0.03^b^	3.49 ± 0.29^b^
Arsenic + FIS	2.42 ± 0.02^a^	3.07 ± 0.04^a^	5.51 ± 0.28^a^
FIS	2.58 ± 0.02^a^	3.19 ± 0.04^a^	6.02 ± 0.05^a^

Data displayed as Mean ± SEM (12 rats/group). Results of each sub-parameter with varying alphabets are considered significantly different (*p* < 0.05).

### Effect of arsenic and FIS on sperm parameters

To determine the alleviative effect of FIS on arsenic-prompted testicular intoxication, the sperm indices were evaluated. Arsenic-intoxicated rats exhibited a decline in the sperm MMP and motility as well as epididymal sperm count and HOS coil-tailed sperms, whereas dead sperms and morphological sperm (head, midpiece and tail) damages were escalated when compared to control (Table 5). However, co-treatment with arsenic and FIS significantly escalated the sperm MMP and motility in addition to the sperm count and HOS-coil tailed sperms, while it abated the dead sperms and morphological sperm (head, midpiece and tail) damages as compared to arsenic-treated rats. Furthermore, FIS-treated rats and control did not have any significant difference.

**Table 5:** Effect of arsenic and FIS on spermatological indices.

Groups	MMP (%)	Motility (%)	Epididymal sperm count (%)	Dead sperms (%)	Head abnormality (%)	Midpiece abnormality (%)	Tail abnormality (%)	Hypo-osmotic swelling test (%)
Control	83.3 ± 1.90^a^	64.5 ± 0.83^a^	28.45 ± 0.65^ab^	18.5 ± 1.37^a^	3.33 ± 0.19^a^	0.78 ± 0.06^a^	4.73 ± 0.09^a^	82.38 ± 2.40^a^
Arsenic	62.4 ± 1.83^b^	31.2 ± 1.16^c^	10.70 ± 0.92^c^	48.4 ± 1.44^c^	8.51 ± 0.16^c^	3.07 ± 0.08^c^	9.82 ± 0.32^b^	24.03 ± 2.89^c^
Arsenic + FIS	56.5 ± 24.2^a^	57.5 ± 0.82^b^	23.76 ± 0.78^b^	26.4 ± 1.19^b^	6.06 ± 0.08^b^	1.19 ± 0.08^b^	4.96 ± 0.04^a^	61.29 ± 3.08^b^
FIS	83.9 ± 1.90^a^	62.25 ± 1.39^a^	29.21 ± 0.83^a^	19.47 ± 0.80^a^	4.02 ± 0.09^a^	0.95 ± 0.03^a^	4.66 ± 0.05^a^	83.24 ± 3.36^a^

Data displayed as Mean ± SEM (12 rats/group). Results of each sub-parameter with varying alphabets are considered significantly different (*p* < 0.05).

### Effect of arsenic and FIS on expressions of apoptotic markers

**Figure 2:**
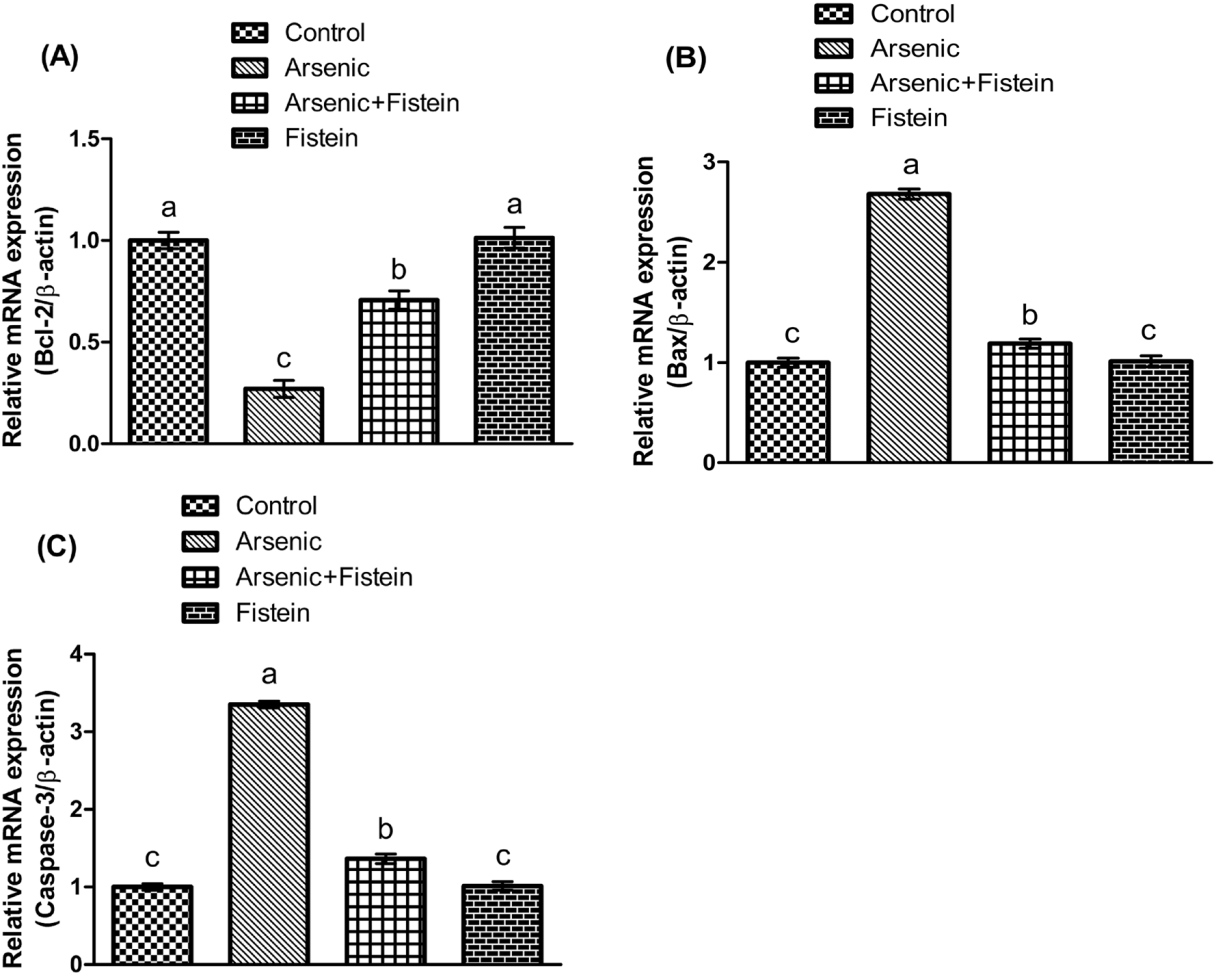
Impact of arsenic and FIS on encoding of of (**A**) Bcl-2, (**B**) Bax, and (**C**) Caspase-3. Data shown as Mean ± SEM (12 rats/group). Means with dissimilar letters vary significantly and the letters indicate significant difference at *p* < 0.05.

To confirm the anti-apoptotic property of FIS, the alterations in gene expressions of apoptotic markers were evaluated. The graphical representations of the varying expressions of these markers are presented in Fig. 2. Exposure of arsenic to the rats remarkably raised the expression of Bax and caspase-3, whereas lessened the expression of Bcl-2 in comparison to control. Conversely, the treatment of arsenic and FIS reversed the expressions of aforementioned apoptotic markers in co-treated group (arsenic + FIS) compared with arsenic-exposed rats. However, there was non-significant difference among the FIS-exposed and control rats.

### Effect of arsenic and FIS on testicular histopathology

**Figure 3:**
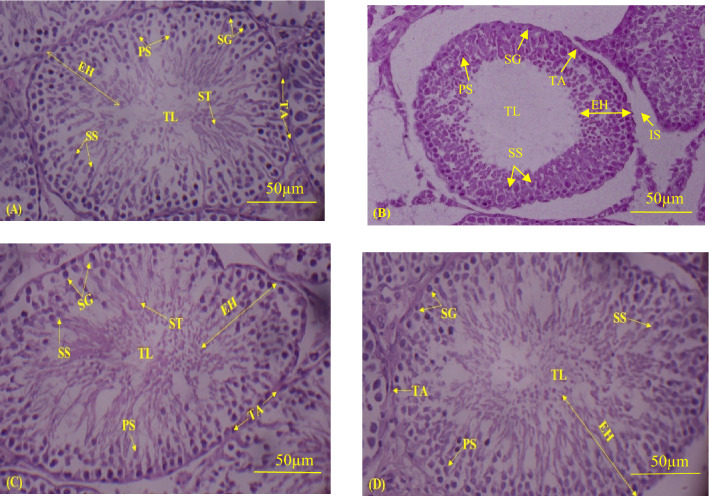
Histoarchitectural changes caused by arsenic and FIS in testes (H&E, 200 ×). (**A**) Control exhibiting thickened epithelium and proper spermatogenesis, (**B**) Arsenic-intoxicated rats showing increased interstitial spaces and degenerated epithelial layer in addition to the luminal space with lowered germ cells number, (**C**) Arsenic + FIS-treated rats presenting restoration in epithelial state, a little reduction in interstitial spaces, and luminal portion with higher number of sperm cells compared with arsenic-administered rats, (**D**) FIS-treated rats showing compact epithelial layer, very less interstitial spaces, and luminal area containing high number of spermatogenic cells. *TA* tunica albuginea, *EH* epithelium height, *TL* tubule luminal area, *IS* interstitium, *ST* spermatids, *PS* primary spermatocyte, *SG* spermatogonia, *SS* secondary spermatocytes.

The testoprotective role of FIS was further validated by histoarchitectural evaluations. Table 6 and Fig. 3 depict the histoarchitectural changes followed by the arsenic and FIS treatment. The administration of environmental toxin, arsenic, significantly escalated the tubular lumen in addition to interstitial spaces, while tunica albuginea thickness, tubular diameter and height of epithelium of seminiferous tubules were significantly (*p* < 0.05) decreased compared to control. Moreover, arsenic reduced the germ cells number when compared to control Nevertheless, the administration of curative compound, FIS, with the arsenic restored these adverse changes in co-administered (arsenic + FIS) group in comparison to arsenic-intoxicated group (Table 6). Moreover, there was a non-significant difference among the FIS-treated and control rats.

**Table 6:** Effect of arsenic and FIS on histopathology of testes.

Groups	Interstitial spaces (µm)	Thickness of Tunica albuginea (µm)	Diameter of seminiferous tubules (µm)	Epithelial height of Seminiferous tubules (µm)	Tubular lumen (µm)	Spermatogonia (n)	Primary spermatocyte (n)	Secondary spermatocyte (n)	Spermatids (n)
Control	6.33 ± 0.08^a^	26.6 ± 0.88^a^	177.3 ± 1.20^a^	76.66 ± 0.88^a^	11.33 ± 1.45^a^	46.66 ± 0.88^a^	41.33 ± 0.45^a^	36.66 ± 1.45^a^	49.66 ± 1.45^a^
Arsenic	12.6 ± 1.45^b^	15.3 ± 1.20^c^	161.3 ± 1.45^c^	33.00 ± 2.08^c^	48.00 ± 1.52^c^	34.33 ± 2.02^b^	27.33 ± 0.27^b^	22.33 ± 1.85^b^	26.66 ± 0.88^c^
Arsenic + FIS	8.00 ± 0.57^a^	22.3 ± 0.88^b^	170.3 ± 1.20^b^	57.66 ± 1.76^b^	20.66 ± 1.20^b^	44.66 ± 0.88^a^	37.33 ± 0.45^a^	31.66 ± 1.45^a^	44.66 ± 0.88^b^
Arsenic	5.33 ± 0.88^a^	28.0 ± 1.15^a^	179.6 ± 0.88^a^	78.66 ± 1.20^a^	09.66. ± 0.88^a^	48.66 ± 1.45^a^	41.66 ± 0.38^a^	35.66 ± 2.02^a^	52.33 ± 0.88^a^

Data displayed as Mean ± SEM (12 rats/group). Results of each sub-parameter with varying alphabets are considered significantly different (*p* < 0.05).

## Discussion

Approximately 200 million people are at the risk of arsenic exposure as well as the arsenic-generated toxicity. Arsenic exposure results in testicular toxicity even at very low concentration^1^. Arsenic reportedly instigates toxicity via arsenolysis, in which binding of sulfhydryl and carbonyl groups with the proteins and replacement of phosphate moieties occur^35,36^. This event ultimately impairs the major phenomena of reproduction system, including steroidogenesis and spermatogenesis, in rats^37^. It is well-documented that the flavonoids are therapeutic agents due to their capacity to bring down oxidative stress owing to their phenolic hydroxyl groups^38^. FIS is reported to confer antioxidative effect via several ways such as, by enzymatic and non-enzymatic antioxidant activities and chelation of metal ions as well as via functioning as the substrate for the oxidoreductase activities^39^. Therefore, in the current study, FIS was administered as a protective agent to attenuate the arsenic-induced reprotoxicity in male rats.

Arsenic administration adversely affected the antioxidant enzymes of rats by decreasing the activity of CAT, SOD, GPx, GSR, and GSH level. CAT is involved in the transformation of hydrogen peroxide into water and oxygen. Moreover, it keeps away the superoxide anions produced by nicotinamide adenine dinucleotide phosphate (NADPH) oxidases from neutrophils and protects sperm cells against the oxidative burden^40^. SOD performs defensive role against damaging impact of oxyradicals in cells by dismutating the superoxide anions into oxygen. Collectively, arsenic exposure is evidenced to bring down the activities of CAT and SOD by reducing the NADH availability and by elevating the generation of superoxide anions^41^. GSH is notably present in several type of cells, which assists in hydrophilic xenobiotic conjugation and further aids in detoxification of toxicants as well as their reactive metabolites. GSH has tendency to bind with the sulfhydryl groups attached with the arsenic, which leads to the sequestration and elimination of this toxin from the tissues. The consumption of GSH during sequestration and elimination of arsenic is the primary reason behind the depletion of GSH content^42^. Whereas, GPx aids in the formation of oxidized glutathione, GSSG (a GSH precursor), and contributes in detoxification^43^. Generally, antioxidative enzymes counter the hazardous effects of ROS. Nevertheless, during the excessive generation of ROS, the body’s antioxidant defence system is unable to neutralize the massive burden of toxic radicals, thereby causing oxidative stress.

Arsenic exposure to the rats resulted in the escalation of TBARS and ROS. It is well-known that TBARS is final product as well as index of lipid peroxidation. As previously mentioned, the spermatogenic membranes are stuffed with PUFAs and therefore, they have tendency to undergo lipid peroxidation^12^. Lipid peroxidation consists of a cascade of free radical-mediated chain reactions, in which peroxidation of membranous lipids distorts the integrity of macromolecules, such as DNA, proteins, nucleic acid and lipids, thereby causing cellular damage^44^. Summing up, the escalation of oxidative burden due to arsenic intoxication was evident by the reduction in activity of CAT, SOD, GPx, GSR, and GSH level. Whereas, the levels of TBARS and ROS were observed to be increased. The current finding is in line with our previous study, in which a decrease in activities of anti-oxidative enzymes, as well increase in lipid peroxidation and ROS were observed following the arsenic exposure^45^. However, the FIS administration escalated the activities of aforementioned antioxidative enzymes and GSH level, in addition to inhibiting the lipid peroxidation by bringing down the levels of TBARS and ROS, thereby confirming its antioxidative and anti-lipoperoxidative potential. It was stated that FIS holds potential to inhibit the peroxidation of lipid by suppressing the free radical penetration into lipid hydrophobic core of cell membranes^46^. FIS possesses o-dihydroxy configuration on B-ring and 3-hydroxy group as well as 2, 3-double bond on C-ring, which enables it to act as a promising antioxidant that scavenges ROS due to its electron-donating capability^47^.

In the present investigation, levels of total cholesterol, triglycerides and LDL were escalated, while HDL level exhibited a decrease in arsenic-intoxicated rats. Oxidative stress leads to the disruption in structure of macromolecules, such as lipids and protein. Lipids play an essential role in structural and functional maintenance of cells and organ systems of body^48^. Hill and Bordoni^49^ stated that the high lipid levels, including cholesterol, LDL and triglycerides lead to a disordered state namely hyperlipidemia, which causes toxic effects in testicles. Additionally, the high level of cholesterol and reduction in level of HDL adversely affect the sperms and decrease the concentration of testosterone, which culminates in male infertility^50^. It was presumed that the disturbance in lipid profile following the arsenic exposure might have occurred due to the arsenic-induced oxidative stress. However, FIS supplementation lowered the level of total cholesterol, triglycerides and LDL, while elevated the HDL level. An earlier investigation indicated that the FIS holds the capacity to protect the tissues from LDL owing to its high lipophilic potential as well as due to its penetrating tendency into the lipid bilayer membrane^51^.

The variations in expressions of steroidogenic enzyme were evaluated to assess the mechanistic approach underlying the lowered level of testosterone following the arsenic intoxication. In the current investigation, the administration of arsenic significantly down-regulated the gene expression of 3β-HSD, 17β-HSD, StAR, CYP11A1 and CYP17A1. Steroidogenesis is the crucial phenomenon for the testosterone synthesis, which is particularly regulated by the steroidogenic proteins and enzymes^52^. Cholesterol acts as a substrate for synthesizing the testosterone. StAR is known to assist in delivering the cholesterol in inner mitochondrial membrane. CYP11A1 has tendency to cleave the cholesterol into pregnenolone in the mitochondria, where the microsomal enzyme, 3β-HSD, alters the pregnenolone to the progesterone. CYP17A1 plays role in the conversion of progesterone into androstenedione. Finally, 17β-HSD transforms the androstenedione into testosterone^53^. It was inferred that lowered level of testosterone following the arsenic exposure is attributed to the reduction in gene expressions of these steroidogenic enzymes. Additionally, it was previously indicated that arsenic has the capability to attach with the aforesaid enzymes, which resultingly affect their performance^54^. However, treating the rats with the phytochemical, FIS, substantially increased the expression of these steroidogenic enzymes owing to the mitigation of oxidative burden as well as its androgenic potential.

In the present study, the hormones of hypothalamus-pituitary–gonadal (HPG) axis were analyzed to examine the role of arsenic on gonadotropins and plasma testosterone. Levels of gonadotropins and testosterone were drastically decreased after the arsenic exposure. Testosterone biosynthesis is one of the major roles of testes for the regulation of spermatogenesis and this hormone is produced by Leydig cells via increased expression of StAR^55^. Lowered level of LH is known to impair the activity of Leydig cells, which leads to the suppression of testosterone^56^. Since FSH is crucial to maintain the testicular functions, including spermatogenesis; therefore, the disturbance in level of FSH directly affects the ultimate objective of male reproductive system i.e., production of sperms^57^. According to Renu et al.^58^, arsenic is evidenced to adversely affect the concentrations of LH and FSH either by causing damage to testes or through suppression of pituitary gland performance. Another study reported that arsenic exhibits estrogenic mode of action, and subsequently suppresses the secretion of gonadotropins^59^. It was inferred that arsenic, being an endocrine disruptor, has tendency to affect the performance of HPG axis, which subsequently causes damages in hormonal levels. However, FIS exposure caused an upsurge in the levels of FSH, LH and plasma testosterone by stimulating the activity of the HPG axis, which eventually restored the detrimental spermatogenic fluctuations toward the normal.

In the current investigation, arsenic exposure decreased the sperm MMP, motility, epididymal sperm count and HOS-coiled tail sperms, while increased the dead sperms and morphological sperm damages. Previous study demonstrated that the nuclear chromatin of sperm cells contains high quantity of thiol-enriched protamines, and flagella of sperms is enriched with thiols; therefore, the male gametes are vulnerable to arsenic-induced damage^60^. The capacity of arsenic to attach with the thiol proteins could affect the structure of protein in flagella on male gametes as well as the suppression of motility-mediating enzymes leads to the lowered sperm motility^61^. Moreover, excessive ROS generation affects internal environment of epididymis as well as results in the peroxidation of PUFAs in sperm membranes, which might be another contributing factor behind reduced motility of sperms^12,62^. Epididymis assists in regulation of forward motility. During the transit of male gametes through epididymis, sperms maturation takes place. However, heavy metals affect the functioning of epididymis, thereby damaging the sperm dynamics^62^. It was reported that one of the forms of ROS, hydrogen peroxide, diffuses through sperm membrane and disturb the activity of enzymes crucial for motility, hence reducing the sperm motility^63^. Furthermore, excessive generation of the ROS causes reduction in intracellular ATP^64^, leading to the decrease in the respiratory rate of mitochondria, which was exhibited by the disturbance in viability, membrane integrity as well as MMP of sperms. MMP is involved in the homeostasis of mitochondria and provides the energy for synthesis of ATP in mitochondria^65^. Moreover, the sperm MMP is regarded as one of the major factors that directly affects the motility, as well as indicates the overall health and performance of sperms and its mitochondria^66^. It was assumed that the reduction in sperm MMP occurred simultaneously and/or prior to the decrease in sperm motility. In addition to the motility, the sperm MMP is shown to directly affect the sperm count as well as structural integrity^67^. Summing up, the above-mentioned arsenic-prompted spermatogenic damages were stimulated due to excessive production of free radicals, and the impairment to each sperm parameter adversely affected the other one. Nevertheless, FIS treatment restored the spermatogenic dynamics to the normal state. Therefore, we inferred that FIS exerts mitigative effects to the sperm dynamics by scavenging ROS and suppressing the lipid peroxidation.

Arsenic intoxication to the rats significantly increased the expression of Bax and caspase-3, whereas decreased the Bcl-2 expression. It was indicated that the arsenic exposure reportedly targets the mitochondria and causes toxicity by causing oxidative stress. Bcl-2 and Bax are considered as anti-apoptotic and pro-apoptotic proteins, respectively and they perform opposing function. Increased level of Bax and decreased level of Bcl-2 affect the permeability of membrane of mitochondria, which subsequently results in leakage of cytochrome-c. The cytochrome-c release leads to activation of caspase as a positive feedback response to promote apoptosis^68^. Pachauri et al.^69^ indicated that caspase proteases are regarded as the contributing proteins, which are revealed to play significant role in inducing the arsenic-generated male reproductive damage. In a recent study, arsenic was observed to induce apoptotic damage in rats by escalating the level of caspase-3^70^. Nevertheless, FIS administration resulted in up-regulation of Bcl-2 expression as well as down-regulation of the expressions of Bax and caspase-3, thereby confirming the anti-apoptotic effect of FIS. It was inferred that FIS played the anti-apoptotic role against arsenic-intoxication probably by regulating the aforementioned apoptotic markers. The present finding is in line with a recent investigation, in which the FIS exposure showed neuroprotective effect by declining the Bax and caspase-3 expression, while raising the Bcl-2 expression in rats affected by spinal cord injury^71^.

Arsenic administration lowered the tunica albuginea height along with tubular height and diameter, whereas increased the luminal diameter of tubules. Furthermore, germ cell count belonging to all four stages was reduced. The current finding of histopathological degeneration of testes following the arsenic intoxication corroborate the results presented in previous study, in which the decrease in diameter of seminiferous tubules, necrosis as well as damage to germ cells were reported^72^. The disruption in steroidogenic activities and the consequent decrease in the level of testosterone is regarded as one of the causative factors underlying the disturbed spermatogenesis^73^. A previous study indicated that the reduction in tubular diameter and epithelial height in rat testes occurred due to the decrease in testosterone production, which also affect the number of sperm cells^74,75^. The degeneration and resultant increase in interstitium probably occurred due apoptotic cell death followed by arsenic exposure. Another study reported that there are several proposed pathways which indicate that arsenic intoxication culminates in the impairment of testicular histology by adversely affecting the testicular tissues via escalation of the oxidative stress^9^. Therefore, we assumed that arsenic-induced increase in oxidative stress and apoptosis, decrease in level of testosterone as well as the damage to the major phenomena of testicular system, such as steroidogenesis and spermatogenesis, contribute to the histological damage of testes. However, FIS treatment exhibited therapeutic potential against the arsenic-generated histopathological damages in male reproductive system. These findings suggested that FIS could mitigate the arsenic-prompted testicular toxicity by attenuating the oxidative stress, thereby indicating its histoprotective potential.

## Conclusion

The current investigation revealed that arsenic escalated the oxidative burden, reduced the activity of antioxidative enzymes and increased the lipid peroxidation. Moreover, it showed a significant uprise in the level of total cholesterol, LDL and triglycerides, whereas a prominent reduction was detected in HDL level. Furthermore, the reduction in expressions of steroidogenic enzymes following the arsenic intoxication brought down the level of testosterone. In addition to it, the level of LH and FSH were drastically lowered. The lowered level of these hormones resulted in the adverse alterations in the all major spermatological parameters including motility, viability, count, sperm morphological anomalies and sperm MMP. Besides, the expressions of apoptotic markers showed alterations and histoarchitectural damages were prompted in testicular tissues. Nevertheless, FIS supplementation significantly (*p* < 0.05) mitigated the abovesaid damages in reproductive system of rats. Therefore, it is proposed that FIS could be used as a therapeutic candidate against arsenic-induced reproductive malfunctions in male rats due to its anti-oxidative, anti-lipoperoxidative, anti-apoptotic, and androgenic efficacy.

## Data Availability

The datasets used and/or analyzed during the current study are available from the corresponding author on reasonable request.

## Abbreviations

ANOVA: Analysis of variance
ATSDR: Agency for Toxic Substances and Disease Registry
CAT: Catalase
CYP11A1: Cholesterol side-chain cleavage enzyme
ELISA: Enzyme-linked immunosorbent assay
FIS: Fisetin
FSH: Follicle-stimulating hormone
GPx: Glutathione peroxidase
GSH: Glutathione
GSSG: Oxidized glutathione
GSR: Glutathione reductase
HDL: High-density lipoprotein
HOS: Hypo-osmotic swelling
HPG: Hypothalamus-pituitary–gonadal axis
HSD: Hydroxysteroid dehydrogenase
LDL: Low-density lipoprotein
LH: Luteinizing-hormone
MMP: Mitochondrial membrane potential
NADPH: Nicotinamide adenine dinucleotide phosphate
PUFAs: Polyunsaturated fatty acids
qRT-PCR: Quantitative real-time quantitative polymerase chain reaction
ROS: Reactive oxygen species
SEM: Standard error of mean
SOD: Superoxide dismutase
StAR: Steroidogenic acute regulatory protein
TBARS: Thiobarbituric acid reactive substance
CYP17A1: 17α-Hydroxylase/17, 20-lyase
